# A new record for a massive *Porites* colony at Ta’u Island, American Samoa

**DOI:** 10.1038/s41598-020-77776-7

**Published:** 2020-12-07

**Authors:** Georgia Coward, Alice Lawrence, Natasha Ripley, Valerie Brown, Mareike Sudek, Eric Brown, Ian Moffitt, Bert Fuiava, Bernardo Vargas-Ángel

**Affiliations:** 1 American Samoa Coral Reef Advisory Group- Department of Marine and Wildlife Resources, Pago Pago, AS 96799 USA; 2 National Marine Sanctuary of American Samoa, Pago Pago, AS 96799 USA; 3 National Park of American Samoa, Pago Pago, AS 96799 USA; 4 Joint Institute for Marine and Atmospheric Science, Honolulu, HI 96822 USA; 5 Cardinal Point Captains, Oceanside, CA 92056 USA

**Keywords:** Ecology, Marine biology

## Abstract

An exceptionally large, hermatypic colony of *Porites* sp. has been identified and measured at Ta’u, American Samoa. This coral was measured in November 2019 as part of an effort to catalogue all large (≥ 2 m diameter) *Porites* colonies around Ta’u. Colonies exceeding 10 m in diameter were recorded on three different sides of the island with seasonally different wave exposures. The largest colony measured 8 m tall, 69 m in circumference and had a diameter of 22.4 m. To date, this is the biggest colony recorded in American Samoa, and one of the largest documented worldwide. It is currently unknown why such large corals exist around this particular island. Possible explanations include mild wave or atmospheric climates and minimal anthropogenic impacts. Physiologically, these colonies may be resistant and/or resilient to disturbances. Large, intact corals can help build past (century-scale) climatic profiles, and better understand coral persistence, particularly as coral communities worldwide are declining at rapid rates.

## Introduction

**Figure 1:**
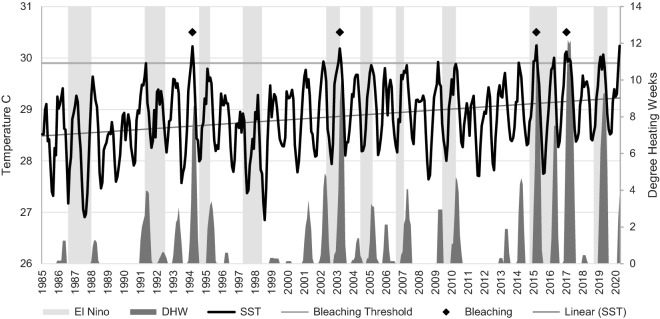
1985–2020 time series of monthly average sea surface temperature (SST). Data is derived from the NOAA Coral Reef Watch virtual station for the Samoan Archipelago^9^ and plotted against bleaching threshold, degree heating weeks (DHW, degree C-weeks), and El Niño events^12^. Degree heating weeks is a measure of accumulated heat stress in reef ecosystems and values greater than 8 C-weeks indicate potential bleaching conditions. Diamonds indicate observed bleaching events. Data was imported and processed in Microsoft Excel 2016.

American Samoa is a small (total area 202 km^2^), unincorporated territory of the United States. Located in the South Pacific, it is comprised of five high islands (Tutuila, Aunu‘u, Ofu, Olosega, Ta’u), one low island (Swains) and an atoll (Rose Atoll). The majority of American Samoa’s population (Census 2010: 55,519) resides on the main island of Tutuila, and at present less than 0.2% inhabit Ofu, Olosega, and Ta’u (Manu‘a Islands). Swains and Rose are currently uninhabited. To date, American Samoa has approximately 250 verified hermatypic coral species^1^ and in general, the reefs have proven fairly resilient to both global and localised stressors such as increasing ocean temperatures^2^ and crown-of-thorns starfish (*Acanthaser* spp.) outbreaks^3^. This resilience has likely been a result of three factors; infrequent acute or short-term disturbance events that can occur on a regular basis in other coral reef environments, such as tropical cyclones and corallivore outbreaks; localised chronic disturbances at small spatial scales (e.g., land-based point sources of pollution) and; the isolation from major land masses^4,5^. However, acute disturbance events, particularly climate related, are increasing in frequency and strength^6–8^. American Samoa experienced severe coral bleaching in 1994, 2003, 2015 and 2017, with several minor/moderate events occurring between 1998 and 2019^8^. Sea surface temperatures (SST) are rising in the region, and local SST now regularly exceed the theoretical bleaching threshold (1 °C above maximum monthly mean SST) during summer months (Fig. 1), increasing the threat of coral bleaching and associated coral mortality^9,10^. El Niño-Southern Oscillation (ENSO) events have had inconsistent effects on local SST and may not be a major driver of coral bleaching in American Samoa^11^. Despite these changes, reefs around the island of Ta’u (14° 13′ 48" S, 169° 27′ 0" W*)* within the Manu‘a Island group continue to thrive and support unique coral assemblages.

The volcanic island of Ta’u is 70,000 years old and lies 107 km east of Tutuila with a total land area of 44 km^2^, and a maximum elevation of 931 m^12^. Brown et al.^13^ previously documented one of the largest, single colonies of massive *Porites* sp. located on the southwest corner of the island. This colony measures approximately 7 m in height, 41 m in circumference, with a diameter of 12 m^13^. The species was identified as *Porites lutea*^13,14^ and core samples taken in 2011 indicate the coral is at least 500 years old^14^. Massive *Porites* colonies in American Samoa consist of a group of species (e.g., *P. lobata, P. evermanni, P. australiensis, P. lutea*), which are mound-shaped and difficult to distinguish in the field. Large colonies have been reported from Ta’u, yet such large *Porites* corals are relatively rare elsewhere. Done and Potts^16^ noted that large massive *Porites* (up to 10 m) on the Great Barrier Reef are scarce and limited to localised aggregations in a specific zone of the reef front terrace (6–12 m depth) or on sheltered inshore reefs. Other locations reporting large *Porites* include Pandora Reef, Great Barrier Reef, (*P. lobata*, 6.85 m diameter, estimated age 677^17^), Green Island, Taiwan (*P. lobata*, 12 m height, 31 m circumference, estimated age 1,000^18^), and Sesoko Island, Japan (*P. australiensis* micro atoll, 11.1 m diameter, 33.7 m circumference, estimated age 500—2,100^19^).

The present study aimed to catalogue all large (≥ 2 m diameter) *Porites* colonies around Ta’u as a multi-agency initiative (Coral Reef Advisory Group Coordination, Department of Marine and Wildlife Resources, National Park Service of American Samoa, National Marine Sanctuary of American Samoa). The study builds upon rapid tow-board surveys conducted in 2008, which identified 32 large massive *Porites* around Ta’u^20^. These surveys were limited and no follow-up research was conducted (B. Vargas-Ángel pers. comm.).

A related survey effort in 2018 identified a single, exceptionally large colony on the eastern side of the island (M. Sudek and B. Vargas-Ángel pers. comm.). Cataloguing the location of such colonies furthers climate science, as these sites have continuously supported corals for hundreds of years. Some hermaptypic corals produce banded aragonite skeletons annually and through coring can provide information on growth rates and age^15^. Cores from long-lived and large corals, coupled with geochemical and isotopic analyses in coral skeletal cores can help understand century-scale changes in ENSO patterns and other oceanographic events, and can be used to verify climate models^15^. Further study of these corals and the reefs that support them may improve our understanding of resilience factors and assist with informing future reef management strategies which can promote the long-term management and protection of these unique areas. This present study aims to expand on the existing evidence and anecdotal reports of exceptionally large *Porites* colonies around Ta’u, American Samoa and to comprehensively document their location and measurements.

## Method

**Figure 2:**
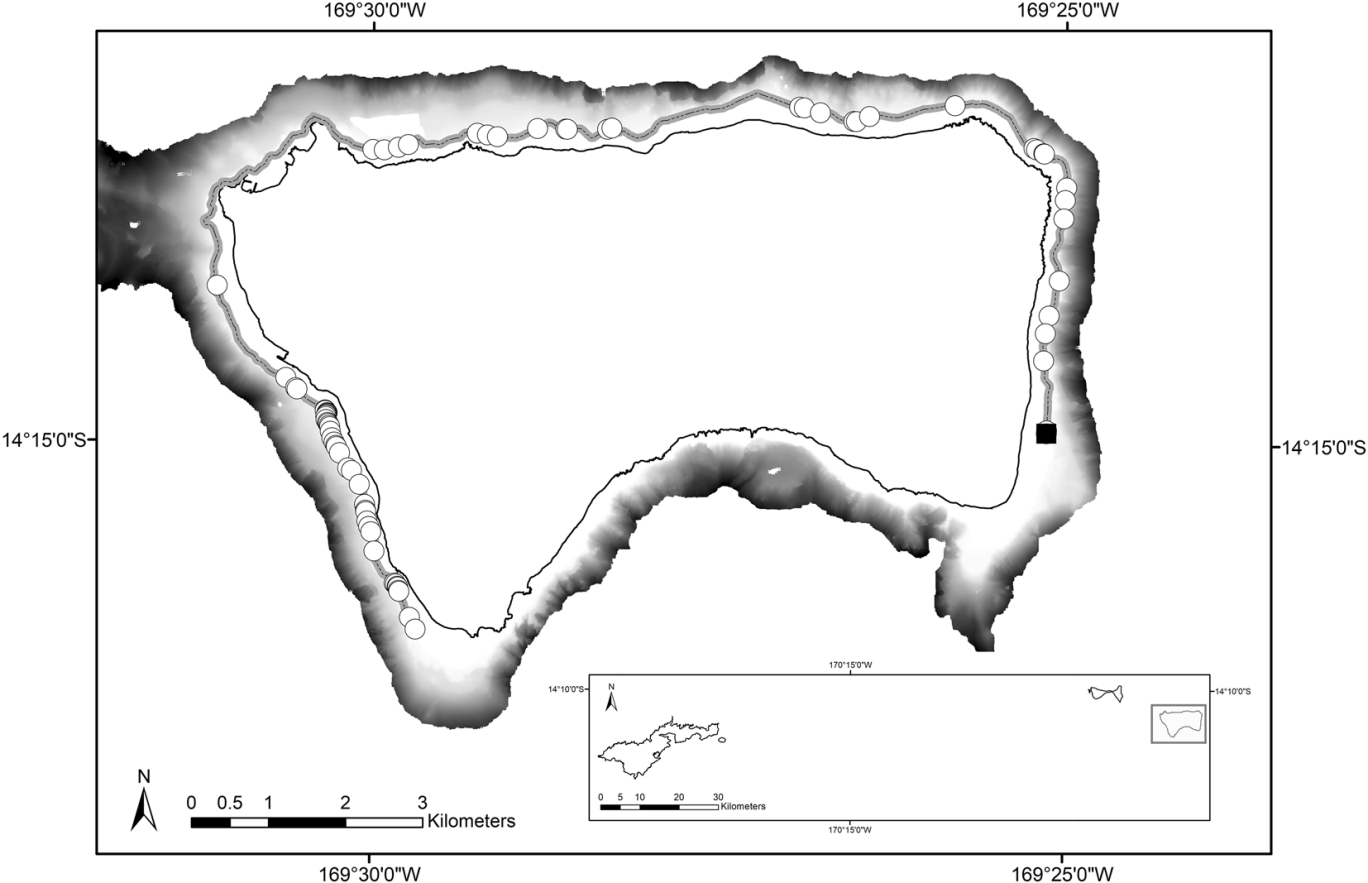
Tow-board survey route (indicated by the black line) around Ta’u, American Samoa and location of colonies of massive *Porites* observed within scale 3 (> 10 m diameter; represented by white circles). The black box designates the location of Ta’u within the Territory and the black square indicates the location of the largest colony observed.

From November 25–27, 2019 tow-board surveys were conducted on the northern, eastern, and western sides of Ta’u (Fig. 2). The southern side of Ta’u was not prioritised due to time constraints and results from previous tow-board surveys that suggested it is an unfavourable habitat for hard coral^20^. The wave exposures around Ta’u are quite distinct given the island’s somewhat rectangular geomorphology. In the austral winter, trade winds hit the east and southern facing shores with the strongest consistent wave energy^10^. The wave energy is composed of short period (~ 2–10 s) seas generated by the local trade winds and longer period (~ 10–20 s) waves originating from far south^21^. Cyclones and other large wave events with more destructive wave power typically strike the Samoan archipelago in the austral summer from the north and northwest, but at infrequent intervals^22,23^.

Surveys were conducted in November due to optimal weather conditions and visibility (> 40 m). Two snorkelers were towed behind an 11.9 m boat and attached to a 6 m rope, at a speed of approximately 1 knot within a depth contour range of 10 to 25 m. The width of the survey area varied with water depth, water clarity, and benthic slope, but was estimated to range from 20 to 30 m for both snorkelers combined. Visibility ranged from 30 to 60 m. All large massive *Porites* corals within visual range and > 2 m in diameter were censused. Massive colonies of *Porites* spp. were scored on a semi-qualitative scale between 1 and 3, where 1 included corals 2–5 m in diameter, 2 included corals 6–10 m diameter, and 3 was assigned to corals with a diameter > 10 m. Colony diameter was used in the categorisation from the surface given the snorkelers’ vertical perspective on the coral colonies and the prevalent use of this metric in the literature. An initial calibration was conducted at the surface for all surveyors using a number of colonies and transect tapes to ensure that surveyors were relatively consistent in their size classifications. Survey pairs were mixed throughout the surveys to reduce observer bias and facilitate calibration between surveyors. The surface support team aboard the towing vessel recorded waypoints, depth, and coral scale using a handheld GPS unit and pre-printed datasheets. Any additional observations of the colony (e.g., partial mortality) were also noted. A GPS track was recorded at 5-s intervals and start and end waypoints for each tow segment were documented.

SCUBA dives were conducted on November 26 and 27, 2019, to document five of the newly catalogued corals recorded during the surveys. These included corals along the eastern, northern, and western exposures. Each coral was photographed and coral colony diameter, circumference and height were measured to the nearest decimeter. Measurements were taken by multiple divers using transect tapes across the widest and tallest distances of the colony. The maximum depths at the base and the top of the colony were noted. General health status was also recorded, including the presence of partial mortality and large growth anomalies. Roving diver surveys were conducted to document species presence, relative abundance of fish, and visual estimates of coral cover at the two largest corals surveyed.

## Results and discussion

**Figure 3:**
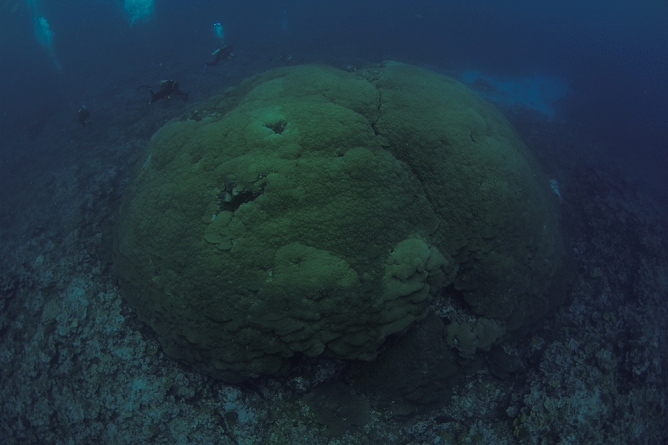
The newly identified and measured *Porites* sp. colony located in Ta’u, American Samoa. Photo credit Alexa Elliott, Changing Seas/South Florida PBS.

Tow-board surveys covered a distance of 21.5 km and the effort identified 84 individual colonies exceeding 10 m in diameter (scale 3; Fig. 2), 393 colonies between 6–10 m (scale 2) and 498 colonies between 2–5 m (scale 1). Table 1 lists the measurements for the five colonies that were documented and measured. The single, largest colony observed was located in 18 m of water and was 22.4 m across, 8 m tall, and the circumference at the base was 69 m (Fig. 3). To date, this is the biggest colony recorded in American Samoa, and one of the largest single colonies documented worldwide. The colony is oval in shape and appears healthy, with an estimated 95% live coral tissue. There are two exposed areas on the top of the colony with a number of small pocilloporid and acroporid colonies. The surrounding habitat appears to be in good condition, with relatively high coral cover and high fish density and diversity. This colony will be named by local communities in Ta’u. Genetic studies are required to confirm that these exceptionally large corals around Ta’u are not multiple colonies merged into one functional unit. Other extremely large colonies of *Porites* spp. have been documented around the globe^18,24^. In some studies, the colonies were reported simply as a matter of record for their unusual size [e.g., 13, 18]. In other cases, colonies were sampled as part of a larger effort to examine growth rates^17^, temperature records coupled with calcification rates^24^, and climate reconstruction^15^. To our knowledge, none of the reported colonies have exceeded the size of the largest colonies measured from Ta’u. A published estimate of growth rates for *Porites* spp. of 19.0 mm year^−1^^25^ in comparable latitudes and adjusted for an average temperature of 28.4 °C near Ta’u^26^, suggest that the two largest corals found on Ta’u are approximately 368 years and 420 years old, respectively. Another age estimate can be generated using the 490 annual density bands measured by Tangri et al.^15^ from a 6.01 m core taken from the coral measuring 7 m in height. This approach yields a linear extension rate of 12.2 mm year^−1^ with age estimates of 571 years for the 7 m coral and 652 years for the 8 m coral. The discrepancy between estimates of over 200 years for both corals is noteworthy and illustrates the variability associated with determining ages for long-lived species.

**Table 1:** Measurements and locations of the largest colonies of massive *Porites* spp. found around Ta‘u, American Samoa in November, 2019. *Previous measurements of a massive *Porites* sp. documented by Brown et al.^13^ are included for comparison.

Date	Latitude	Longitude	Diameter (m)	Height (m)	Circumference (m)
26/11/2019	− 14° 14′ 55.0"	− 169° 25′ 8.4"	22.4	8.0	69.2
11/04/2009*	− 14° 15′ 00.0"	− 169° 30′ 00.0"	17.0	7.0	41.0
27/11/2019	− 14° 14′ 53.2"	− 169° 30′ 18.0"	9.2	4.6	26.3
26/11/2019	− 14° 12′ 37.8"	− 169° 26′ 56.4"	5.7	5.0	25.6
27/11/2019	− 14° 14′ 38.0"	− 169° 30′ 32.4"	5.1	4.3	25.2
27/11/2019	− 14° 14′ 37.7"	− 169° 30′ 32.4"	7.6	4.6	23.4

One of the most interesting aspects of this study is that multiple massive *Porites* colonies were documented on three sides of the island, where they are subjected to differing environmental regimes (e.g., wave exposure, wind exposure, ocean current strength, and direction) that may affect growing conditions. As noted earlier, corals on the eastern and south facing shorelines experience the strongest wave energy on average^10^. In contrast, the generally calmer conditions on the northern and western shores are infrequently impacted by acute cyclonic events^22^. The number of colonies per linear kilometer along each coastline of Ta’u indicated that even though large colonies were found on three sides of the island, there were a disproportionately higher number of the largest scale 3 colonies on the west coast (64%) compared to the north (14%) and east coasts (22%). This pattern was similar for the two smaller size classes (scale 2–53% W, 27% N, 20% E; scale 1–46% W, 38% N, 16% E). These distributional patterns can be partially explained by the wave shadow created by Ofu and Olosega that protect Ta’u from the largest waves coming from 315° to 0°^23^. Wave events originating from 220° to 300° were almost non-existent from 1980 to 2011^23^.

The impact of currents on various coastlines is somewhat more complicated. At a regional scale, currents primarily flow from east to west through the archipelago in the South Equatorial Current, but can reverse directions in the South Equatorial Counter Current depending on the season and/or year^27,28^. Consequently, the oligotrophic open ocean water that would typically influence the eastern shoreline could also periodically appear on the western shoreline. At the local scale, the angular coastline of Ta’u could generate eddies that produce mixing zones that entrain coral larvae and nutrients on all coastlines, but these studies have not yet been conducted.

Colonies were also located across a wide depth gradient from 9 m to over 30 m. Done and Potts^16^ found some aggregations of large *Porites* colonies > 10 m in diameter on exposed, mid-shelf reefs in the Great Barrier Reef, but these colonies were largely confined to the base of the reef slopes (~ 6 to 12 m deep) and they did not appear to approach the size and abundance of the colonies around Ta’u. At this time, it is unknown why so many large coral colonies exist around this particular island. Possible explanations include relatively stable oceanic conditions^22^, which enables these corals to avoid major disturbances (e.g.^29,30^), and minimal anthropogenic impacts that have allowed these corals to survive over centuries. For example, Tangri et al.^15^ suggested that long-lived corals around Ta’u may have benefited from the island’s location in and near the ENSO null zone over the last five centuries. The null zone is theorised to dampen or mute changes in SST related to ENSO events that have been documented in other regions of the Pacific^31^. SST from 1870 to 2019 near Ta’u (− 14° 30′ S, − 169° 30′ W) showed higher average temperatures, but slightly lower inter-annual variation (mean: 28.49° C ± 0.72 SD) compared to temperatures near the Great Barrier Reef (26.66° C ± 0.18 SD) at the same latitude (− 14° 30′ S, 144° 30′ E). The coefficient of variation for the Ta’u temperature measurements was 62% less than the corresponding temperature readings for the GBR values^25^. Hence, these conditions may have allowed the Ta’u corals to continue growing when massive *Porites* corals at other locations, such as Rangiroa, French Polynesia, the Great Barrier Reef, Australia, and Jarvis Island, US Pacific Remote Islands, slowed growth or perished in bleaching events^32–34^.

Post-bleaching core samples of long-lived *Porites* in Jarvis Island revealed that colonies ceased their growth during bleaching events and resumed once normal thermal conditions re-established^34^. This physiological shift enabled these massive *Porites* coral colonies to survive disturbance events such as bleaching and illustrated the resilience of these corals over long time periods within the context of a changing climate. Previous studies such as Loya et al*.*^35^ and Edmunds et al*.*^36^, have suggested that massive *Porites* are more physiologically resilient to disturbances compared to other coral species. It is likely that the corals around Ta’u are utilising similar physiological mechanisms to achieve their large size.

Identifying areas where long-lived corals exist and thrive will be critical in evaluating changes in climate patterns, and identifying resilience factors that may improve management of coral reef ecosystems in the South Pacific. Due to the large number of these massive colonies, it is evident that the island of Ta’u has ideal conditions that support these resilient corals and this warrants some level of protection.
